# Epigenetic Modifications to H3K9 in Renal Tubulointerstitial Cells after Unilateral Ureteric Obstruction and TGF-β1 Stimulation

**DOI:** 10.3389/fphar.2017.00307

**Published:** 2017-05-29

**Authors:** Timothy D. Hewitson, Stephen G. Holt, Sven-Jean Tan, Belinda Wigg, Chrishan S. Samuel, Edward R. Smith

**Affiliations:** ^1^Department of Nephrology, The Royal Melbourne Hospital, MelbourneVIC, Australia; ^2^Department of Medicine, The Royal Melbourne Hospital, The University of Melbourne, MelbourneVIC, Australia; ^3^Cardiovascular Disease Program, Biomedicine Discovery Institute and Department of Pharmacology, Monash University, MelbourneVIC, Australia

**Keywords:** epigenetics, fibroblast, fibrosis, kidney, myofibroblast, histone, TGF-β1, proximal tubule

## Abstract

**Introduction:** Epigenetic regulation of fibrogenesis through post-translational histone modifications (marks) may be a key determinant of progression in renal disease. In this study, we examined the distribution and acquisition of histone 3 Lysine 9 (H3K9) marks after injury and stimulation with the pro-fibrotic cytokine TGF-β1. Our focus was on their presence in activated fibroblasts (myofibroblasts) and epithelial cells (epithelial-mesenchymal transition).

**Methods and Results:** Immunofluorescent microscopy was used to examine global H3K9 acetylation (H3K9Ac) and tri-methylation (H3K9Me3) after unilateral ureteric obstruction (UUO) in mice. Confocal, super resolution microscopy and flow cytometry were used to determine the *in vitro* effect of TGF-β1 on structural arrangement of these marks, and their relationship with α-smooth muscle actin (αSMA) expression, a marker of myofibroblasts and early EMT. The number of individual histone marks was increased 10 days after UUO (*p* < 0.05 vs. control), with both marks clearly seen in various cell types including proximal tubules and myofibroblasts. Sub-nuclear microscopy in primary rat renal fibroblasts and a proximal tubule cell line (NRK-52e) showed that H3K9Ac was co-localized with phosphorylated-Ser2 RNA polymerase II (pRNAPol II), while H3K9Me3 was not, consistent with permissive and repressive effects on gene expression respectively. In both cell types H3K9Ac was diffusely distributed throughout the nucleus, while H3K9Me3 was found in compartments resembling the nucleolus, and in the case of the fibroblast, also juxtapositioned with the nuclear membrane. TGF-β1 had no effect on H3K9Ac marks in either cell, but resulted in a redistribution of H3K9Me3 within the fibroblast nucleus. This was unrelated to any change in mitogenesis, but was associated with increased αSMA expression.

**Conclusion:** These findings highlight why it is important to consider the epigenetics of each cell individually, because whilst no overall enrichment occurred, renal myofibroblast differentiation was accompanied by distinct changes in histone mark arrangements.

## Introduction

After acute renal injury occurs, normal tissue repair mechanisms are often able to restore kidney function. However, if such mechanisms are disrupted, the injury is severe, or the cause of the injury persists, acute kidney injury can progress into a chronic disorder characterized by non-recoverable organ remodeling and scarring (fibrosis) (Hewitson, 2009). Renal parenchymal fibrosis therefore represents the final common pathway in all progressive chronic kidney disease (CKD).

Histologically this process manifests itself as glomerular and vascular sclerosis and tubulointerstitial fibrosis, with the last of these the best predictor of disease progression (Hewitson, 2009). The interstitial fibroblast and tubular epithelial cell are therefore major effector cells in this process. Activation of these cells by paracrine and autocrine signals is responsible for a prodigious production of extracellular matrix, in particular the collagens that constitute scar tissue. In each case activation can be recognized by *de novo* expression of α-smooth muscle actin (αSMA), a marker of myofibroblast differentiation in fibroblasts, and epithelial-mesenchymal transition (EMT) in renal tubule epithelial cells (Hewitson, 2009).

Since its first mechanistic description in the kidney nearly 30 years ago (Border and Ruoslahti, 1990), transforming growth factor -β1 (TGF-β1) is widely considered to be the preeminent fibrogenic cytokine with a multiplicity of evidence showing that it enhances fibroblast recruitment, activation and fibrogenesis both *in vivo* and *in vitro* (Meng et al., 2016). Specific cellular targets of TGF-β1 in kidney disease include fibroblasts, mesangial cells, and tubular epithelial cells amongst others. We also increasingly recognize that such differentiation pathways are not only directly regulated through paracrine signals, but also indirectly regulated through epigenetic mechanisms which integrate pro-fibrotic signals and fibrogenesis (Reddy and Natarajan, 2015).

Epigenetics refers to changes in gene activity that are stable and can be inherited over cycles of cell division, but do not involve changes in the DNA sequence. Euphemistically termed a second genetic code, epigenetic regulation of gene expression in the kidney, as elsewhere, includes RNA interference, post-replicative DNA methylation, and histone modifications (Wing et al., 2013). Although DNA methylation (Bechtel et al., 2010), and miRNA regulation (McClelland et al., 2015; Gomez et al., 2016) of kidney fibrosis have received much attention, post-translational histone modifications (marks) in nucleosomes are poorly understood.

Nucleosomes, the building blocks of chromatin, are made up of chromosomal DNA wrapped around four core histone subunits (H2A, H2B, H3, and H4). Each histone has a flexible N-terminus (tail) that protrudes from the nucleosome. These amino acids are subject to post-translational modifications including phosphorylation, sumoylation, ubiquitination, acetylation (Ac) and methylation, the last being either monomethylation (Me), dimethylation (Me2) or trimethylation (Me3). Certain Lysine (K) amino acids, for example the 9th Lysine (K9) in the histone 3 tail (H3K9), are known to be particularly prone to modification. H3KAc is generally associated with relaxed chromatin and active gene expression. On the other hand, H3K methylation can serve as an active or repressive mark depending on the Lysine residue modified and the extent of methylation. These histone modifications serve as docking sites for coactivators, co-repressors, chromatin remodeling proteins, and various other proteins. The combined effects of the various histone marks form a ‘histone code’ that regulates transcription (Allis and Jenuwein, 2016).

Histone mark regulation of fibroblast-like differentiation is recognized in a multiplicity of pathologies including liver (Mann et al., 2010; Perugorria et al., 2012), nasal mucosa (Cho et al., 2012), cornea (Zhou et al., 2010), lung (Guo et al., 2009), and kidney (Irifuku et al., 2016) fibrosis. Consistent with this, TGF-β1 has been shown to enrich various active histone marks and decrease levels of repressive marks at pro-fibrotic gene promoters in renal mesangial cells (Sun et al., 2010; Yuan et al., 2013), epithelial cells (Sasaki et al., 2016), and fibroblasts (Irifuku et al., 2016; Sasaki et al., 2016). However, while these studies have clearly delineated functional significance, the emphasis has been on specific gene transcription.

To investigate the changes in phenotype during differentiation further we used a combination of *in vivo* and *in vitro* techniques to examine the distribution of histone marks in experimental progressive fibrosis, and how these are regulated by TGF-β1. In our study we have emphasized the effects on cell differentiation and the morphological changes in nuclear architecture that accompany this process. We were particularly interested in Lysine 9 on histone 3 (H3K9), as it can be both acetylated and methylated (Allis and Jenuwein, 2016), and H3K9Ac (Yuan et al., 2013) and H3K9Me3 (Sun et al., 2010; Perugorria et al., 2012) have been directly implicated in fibrogenesis.

## Materials and Methods

### Animal Model

Male C57Bl6 mice were obtained from Monash Animal Research Platform (Melbourne, VIC, Australia) and maintained under normal housing conditions with a 12 h light/12 h dark lighting cycle and free access to food and water. After a 4–5 day acclimatization period unilateral ureteric obstruction (UUO) was performed in mice (aged 8–10 weeks) under inhalational general anaesthesia (Methoxyflurane, Abbott, Sydney, NSW, Australia). Eighteen animals were randomly allocated to 3 or 10 days of UUO or a non-UUO control group (*n* = 6 for each group). In the case of UUO, the left ureter of each animal was obstructed with a 0.4–1.0 mm microvascular clamp (S&T, Neuhausen, Switzerland), while the contralateral ureter was left intact. The incision was sutured and mice allowed to recover with Temgesic (Burprenorphine; Reckitt Benckiser, West Ryde, NSW, Australia) administration. These experiments were approved by the Monash University Institutional Animal Ethics Committee (MARP/2015/011), which adhere to the *Australian Code of Practice for the Care and Use of Laboratory Animals for Scientific Purposes.*

At 3 days (D3; when fibrogenesis occurs) and 10 days (D10; when fibrosis is established) post-UUO, animals were sacrificed by anaesthetic overdose and kidney tissue rapidly excised and cut into transverse sections (each containing cortex and medulla) for immersion fixation in methyl carnoy’s or 4% paraformaldehyde in phosphate buffered saline (PBS). A parallel control group consisted of tissue taken from control non-UUO animals (D0).

### Immunohistochemistry

Immunoperoxidase staining was used to qualitatively examine myofibroblast recruitment (αSMA staining), and collagen I and IV deposition before and after UUO, as described previously (Hewitson et al., 2010). In brief, sections of 4% paraformaldehyde fixed, paraffin embedded tissue were labeled with mouse anti-αSMA (Dako, Glostrup, Denmark) conjugated to biotin (ARK kit; Dako). Labeling was visualized with 3′-diaminobenzidine (DAB; Dako) and hematoxylin counterstaining. Similarly, prepared methyl carnoy’s fixed sections were stained with goat-anti-collagen IV (Southern Biotechnology, Birmingham, AL, United States) or rabbit anti-collagen I (Biodesign International, Saco, ME, United States). Unconjugated antisera were detected with biotinylated anti-IgG secondary antibody, and biotin amplified with avidin-biotin-complex (ABC Elite; Vector, Burlinghame, CA, United States). Again labeling was visualized with DAB (Dako) and hematoxylin.

### Immunofluorescence Staining

For staining of histone marks, sections of tissue fixed in 4% paraformaldehyde and embedded in paraffin were dewaxed, boiled under pressure in citrate buffer (pH 6.0), and equilibrated in PBS for 30 min. Non-specific binding sites were blocked with 10% goat serum (Vector) in 3% bovine serum albumin (BSA)/PBS (pH 7.6) containing 0.1 M glycine for 1 h at room temp (RT). Slides were then incubated with rabbit monoclonal anti- H3K9Ac (Abcam, Cambridge, United Kingdom; cat# 61231) or anti-H3K9Me3 (Abcam; Cat# 8898) in 1% BSA in PBS overnight at 4°C. For co-staining of histones and αSMA, paraformaldehyde fixed sections were treated as above, before being incubated simultaneously with monoclonal anti-αSMA (Dako) and antibodies to individual histone marks in1% BSA/PBS for 2 h at RT. Binding was visualized by addition of Alexa Fluor 488 anti-rabbit IgG, Alexa Fluor 647 anti-rabbit IgG, and/or Alexa Fluor 594 anti-mouse IgG secondaries (Life Technologies, Carlsbad, CA, United States) in 1% BSA/PBS for 2 h at RT, as appropriate. In some cases sections were then incubated with fluorescein labeled Lotus tetragonolobus lectin (LTL) (Vector) in 1% BSA in PBS for 2 h at RT after secondary antibodies to identify proximal tubule brush borders. DAPI (2 μg/mL in PBS, 15 min, RT) was used as a nuclear stain. Finally, sections were washed in PBS and mounted in Vectashield Hard Set (Vector). Low power images were taken at 20x magnification using a Zeiss AXIOSKOP2 microscope (Carl Zeiss, Oberkochen, Germany), or visualized on a Leica SP5 confocal microscope (Leica, Buffalo Grove, IL, United States) with a 63x oil objective for high power imaging.

### Cell Culture and Treatments

Primary banked cell cultures of fibroblasts propagated from fibrotic kidneys (3 days after UUO) of Sprague-Dawley rats were utilized for these studies (Grimwood and Masterson, 2009). Cultures were maintained in Dulbecco’s modified Eagle Medium (DMEM; Sigma) supplemented with 10% fetal calf serum (FCS; Biocore, Melbourne, VIC, Australia), 2.2% HEPES, 1% L-glutamine, penicillin (50 U/mL) and streptomycin (50 μg/mL) (all Sigma) in a humidified incubator at 37°C and 5% CO_2_. For experimental work, cells were seeded into 6-well plates (Costar, Corning, NY, United States) at 1 × 10^6^ cells/well for Western blotting or in 25 cm^2^ flasks (Costar) at 5 × 10^6^ cells/flask for flow cytometric analysis. After attachment overnight and removal of floating cells, fibroblasts were typically cultured for a further 24–48 h in maintenance growth medium before switching to FCS-reduced media (1% FCS) for 24 h before experiments.

NRK-52e cells were obtained from the American Type Culture Collection (Manassas, VA, United States) and maintained in DMEM, 10% FBS and supplements as above. Cells were cultured at 37°C in a humidified atmosphere of 5% CO_2_ in air and passaged twice a week. Cells were seeded in 6-well culture plates. Near confluent NRK-52e cells were subsequently transferred to serum-free Opti-MEM (Sigma) for overnight starvation prior to each experiment.

Fibroblasts and NRK-52E were treated with 1 and 10 ng/ml TGF-β1 (PeproTech, Rocky Hill, NJ, United States) in DMEM/5% FCS respectively for 48 h. In the control groups, cells were treated with DMEM/5% FCS only. The TGF-β1 dose administered was based on that previously shown to maximally stimulate αSMA expression in (myo)fibroblasts (unpublished observations) and NRK-52e (Doi et al., 2011).

### Immunocytochemistry

Fibroblasts and NRK-52e were seeded onto glass coverslips (Carl Zeiss) in 6 well plates at low density (1 × 10^5^/well), and treated with 5%FCS/DMEM or 5%FCS/DMEM with TGF-β1 (PeproTech) for 48 h. After treatment, coverslips were washed with warm DMEM, and cells simultaneously fixed and permeabilised in 4% paraformaldehyde 0.2% Triton X-100 in PBS for 10 min at RT. After washing in PBS, cells were post-fixed in 4% paraformaldehyde in PBS for 5 min. Cells were then blocked in 10% normal goat serum/3% BSA/0.1 M glycine in PBS for 45 min. For staining, coverslips were incubated with primary antibodies (in isolation or combination) diluted in blocking buffer for 2 h at RT in a humidified chamber, washed three times in PBS (5 min each), and then incubated in Alexa Fluor-conjugated goat anti-species secondary antibodies (Life Technologies) diluted in blocker for 60 min in the dark at RT. Primary antibodies used were mouse anti-αSMA (Dako), rabbit anti-vimentin (Abcam), mouse anti-E-cadherin (BD Biosciences, San Jose, CA, United States), rabbit anti-H3K9Ac (Abcam; Cat#61231), rabbit anti-H3K9Me3 (Abcam; Cat# 8898), anti-phosphorylated RNA polymerase II (pSer2 RNA pol II; Abcam) and mouse anti-nucleoporin 62 (NUP62; BD Biosciences). Nuclear staining of DNA was performed with DAPI (1 μg/mL diluted in PBS) for 15 min at RT. Finally, coverslips were washed three times in PBS (5 min each) and mounted in Vectashield HardSet (Vector). Low power images were captured with Zeiss AXIOSKOP2 microscope (Carl Zeiss) for cell characterization, while high power images for cytoskeletal and nuclear morphology were prepared using a Leica SP5 confocal microscope using a 63x oil immersion objective. In a separate experiment, super-resolution OMX blaze microscopy (DeltaVision, GE Healthcare, Pittsburg, PA, United States) was used to examine simultaneous fluorescent labeling of H3K9Me3 and NUP62 in fibroblasts.

For initial characterization studies, coverslips of NRK-52e were also prepared as above and incubated with the proximal tubule specific biotinylated lectin Phaseolus hemaggutinin-l (Pha-L) (Vector), or mouse anti- vimentin (Abcam) followed by a biotinylated secondary anti-mouse antibody (Vector). Labeling was visualized using an ABC Elite kit (Vector), DAB (Sigma), with hematoxylin counterstaining.

### Image Deconvolution and Volume Rendering

Confocal and OMX 3D image stacks were directly imported into Huygens Professional (Scientific Volume Imaging, Hilversum, Netherlands) for deconvolution and volume rendering. All images were deconvoluted using a theoretical point spread function for each channel, and the classical likelihood estimation algorithm. The signal to noise ratios and background intensities were automatically determined and taken into account during processing.

### Morphometrics

To quantify the presence of histone marks in tissue sections, a minimum of three micrographs for each section were taken using a x20 objective (Zeiss AXIOSKOP2) in a blinded fashion, at equivalent settings. Fiji-Image (Schindelin et al., 2012) was then used to quantitate the number of histone marks in each field (0.25^2^ mm) by thresholding. A similar analysis of DAPI staining at each time point was used as a measure of cellularity.

For co-localisation of signal intensity in individual nuclei, we manually plotted a line at the central nuclear plane. The “plot profiler” function of Fiji-Image was used to measure signal intensity of each fluorochrome channel across the length of that line. Individual signal intensities were normalized and plotted on the same axis using GraphPad Prism (GraphPad software, La Jolla, CA, United States).

### Western Blotting Analysis

Western blot analysis with previously described techniques (Hewitson et al., 2016) was variously used to determine changes in αSMA, the epithelial cell transmembrane junction protein E-cadherin, and individual histone marks, in (myo)fibroblasts and NRK-52e.

Protein samples were isolated from cell cultures using RIPA (Sigma), with total protein concentration determined by the BCA assay (Thermo Scientific, Rockford, IL, United States). Histone extracts were isolated from cells using Histone Extraction Kit (Abcam). Briefly, harvested cells were resuspended in pre-lysis buffer and placed on ice for 10 min, spun 9,300 *g* for 1 min, before being resuspended in lysis buffer and incubated on ice for 30 min. After a further centrifugation (5 min, 4°C, 13,400 *g*) the supernantant fraction was collected, Balance-DTT buffer was added and the sample was stored at -80°C until analysis. Proteins (5–20 μg/lane) were separated on 10% Mini-PROTEAN TGX stain free pre-cast gels (Bio-Rad, Hercules, CA, United States) and transferred onto PVDF using the Trans-Blot Turbo transfer system (Bio-Rad). Non-specific protein binding sites were blocked with BLOTTO (Thermo Scientific) for 2 h at RT before being probed with mouse anti-αSMA (Dako), mouse anti-E-cadherin (BD Biosciences), rabbit anti H3K9Ac (Abcam; Cat# 61231), or rabbit anti-H3K9Me3 (Abcam; Cat# 8898) antibodies in incubation buffer (5% BSA supplemented with 0.1% Tween-20 in TBS) overnight at 4°C with agitation. Membranes were rinsed the following day in TBS containing Tween-20 (Bio-Rad). Primary antibody binding was identified with rabbit HRP-conjugated anti-IgG specific for the primary antibody (Abcam), and visualized using SuperSignal West Dura chemiluminescence reagent (Thermo Scientific). Following development, blots were re-probed for β-tubulin (Abcam) or anti-H3 (Cell Signaling Technology, Danvers, MA, United States), as appropriate, to confirm equivalent loading.

### Flow Cytometric Analysis

Flow cytometric analysis was used to identify changes in epigenetic cell states (Obier and Muller, 2010) and corresponding changes in αSMA expression simultaneously.

Intracellular staining of H3K9Ac, H3K9Me3, and αSMA were performed using standard protocols for indirect detection. Briefly, cells were detached from culture vessels using trypsin, and pelleted with centrifugation (x300 *g*). Cells were resuspended in 1 ml of flow cytometry staining buffer (eBioscience, Thermo Scientific), spun for 5 min at 300 *g*, with the pellet resuspended in 100 μl of BD Cytofix/Cytoperm Fixation/Permeabilisation solution (BD Biosciences). After incubation on ice for 30 min, 1 ml of BD Perm/Wash buffer (BD Biosciences) was added, and cells spun for 5 min at 300 *g*. The pellet was resuspended in 200 μl of 10% DMSO in FCS (v/v) before storage at -80°C for subsequent batched analysis. At analysis, aliquots (2 × 10^5^ cells) were incubated with antibodies specific to each mark for 60 min at 4°C. After washing, cells were resuspended in staining buffer containing fluorochrome-labeled secondary antibodies and incubated for 30 min at 4°C in the dark. Cells were then washed and resuspended in ice-cold staining buffer for analysis. Isotype controls were from Abcam (rabbit monoclonal IgG; mouse monoclonal IgG; rabbit polyclonal IgG). For αSMA intracellular staining, fibroblasts were detached with Trypsin/EDTA and incubated with viability dye as described above. Cells were fixed with BD Cytofix fixation buffer (BD Biosciences), permeabilised with BD Phosflow Perm Buffer III (BD Biosciences), and stained with Alexa Fluor 647 rabbit monoclonal anti-αSMA (Abcam) for 30 min at RT in the dark. Cells were washed in Perm/Wash buffer (BD Biosciences) and analyzed on a BD FACSVerse (BD Biosciences) alongside cells stained with appropriate isotype control (Alexa Fluor 647 rabbit monoclonal IgG; Abcam).

### Cell Cycle Analysis

Cell cycle analysis by flow cytometry with a DNA intercalating dye (propidium iodide) was used to measure the frequency of cells in the G0/G1 phase of the cell cycle. Cells were harvested with trypsin, washed twice with PBS, fixed in cold 70% (vol/vol) ethanol, and stored at 4°C until use. Before flow cytometric analysis, cells were washed with PBS and centrifuged, and the cell pellets were resuspended in a solution of RNAse (1 mg/ml) and propidium iodide (80 μg/ml) in PBS for 30 min. Stained cells were analyzed with a BD FACS Verse (BD Bioscience) flow cytometer. For each group, the software was used to calculate the proportion of cells in the G0/G1 phase of the cell cycle.

### Statistical Analysis

Data are expressed as individual data points or mean ± SD, as indicated. Results were analyzed by one-way ANOVA, using the Newman–Keuls and Sidak *post hoc* tests to correct for multiple comparisons between groups. Two-tailed *p* < 0.05 was considered statistically significant.

## Results

### *De novo* Accumulation of Myofibroblasts and Progression of Fibrosis in Obstructive Uropathy

**FIGURE 1:**
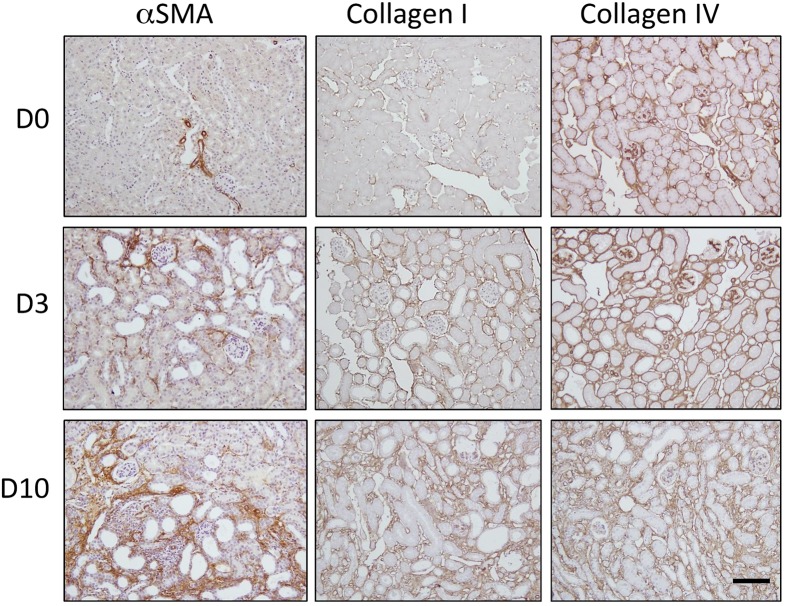
**Progression of fibrosis in the mouse after unilateral ureteric obstruction (UUO)**. Immunohistochemical staining for αSMA (a marker of myofibroblast recruitment) and the extracellular matrix proteins collagen I and collagen IV. Representative micrographs are shown from normal kidneys (D0) and kidneys 3 days (D3) and 10 days (D10) post-UUO. Nuclei were counterstained with hematoxylin. Scale bar = 100 μm.

Representative micrographs are shown from normal kidneys (D0) and kidneys 3 days (D3) and 10 days (D10) post-UUO (**Figure 1**). In the normal kidney, αSMA staining was confined to the vasculature. Collagen I and collagen IV were restricted to the interstitium and basement membranes respectively. Consistent with the well-described natural history in this model (Chevalier et al., 2009), UUO resulted in *de novo* interstitial staining for αSMA, a marker of myofibroblast recruitment. This was paralleled by a rapid and progressive tubulointerstitial increase in collagen subtypes normally associated with the interstitium (collagen I) and the tubule basement membrane (collagen IV).

### Histone H3 Lysine 9 Acetylation (H3K9Ac) and Trimethylation (H3K9Me3) Before and After UUO

**FIGURE 2:**
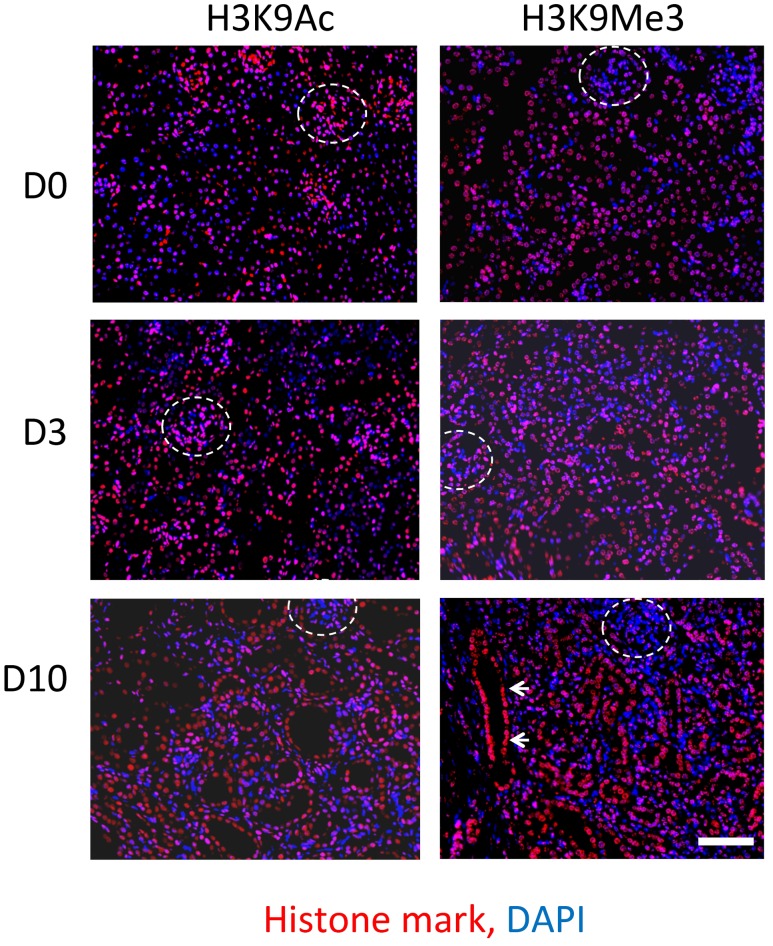
**Immunofluorescent staining of H3K9 acetylation (H3K9Ac) and tri-methylation (H3K9Me3) before and after UUO**. Comparison of staining in normal mouse kidney (D0) and 3 days (D3) and 10 days (D10) after UUO. Merged images show respective histone marks (red) and nuclear DNA with DAPI (blue). Glomeruli are outlined by white dotted lines. White arrows highlight increased intensity in a dilated tubule. Scale bar = 100 μm.

Immunofluorescent staining was used to compare the spatial distribution of Lysine 9 acetylation (H3K9Ac) and trimethylation (H3K9Me3) in both the normal kidney, and after UUO (**Figure 2**). Both marks were widely distributed in the tubulointerstitium of D0 kidneys and after UUO. Likewise both marks were found in glomeruli at all time points although H3K9Me3 marks were less common and intense than H3K9Ac. Visually at least, the intensity of H3K9Me3 staining in areas of overt pathology appeared greater 10 days after UUO. Whilst both histone marks were diffusely distributed, merged immunofluorescent staining for histone marks and DAPI showed that marks were not present in all cells at any time point.

**FIGURE 3:**
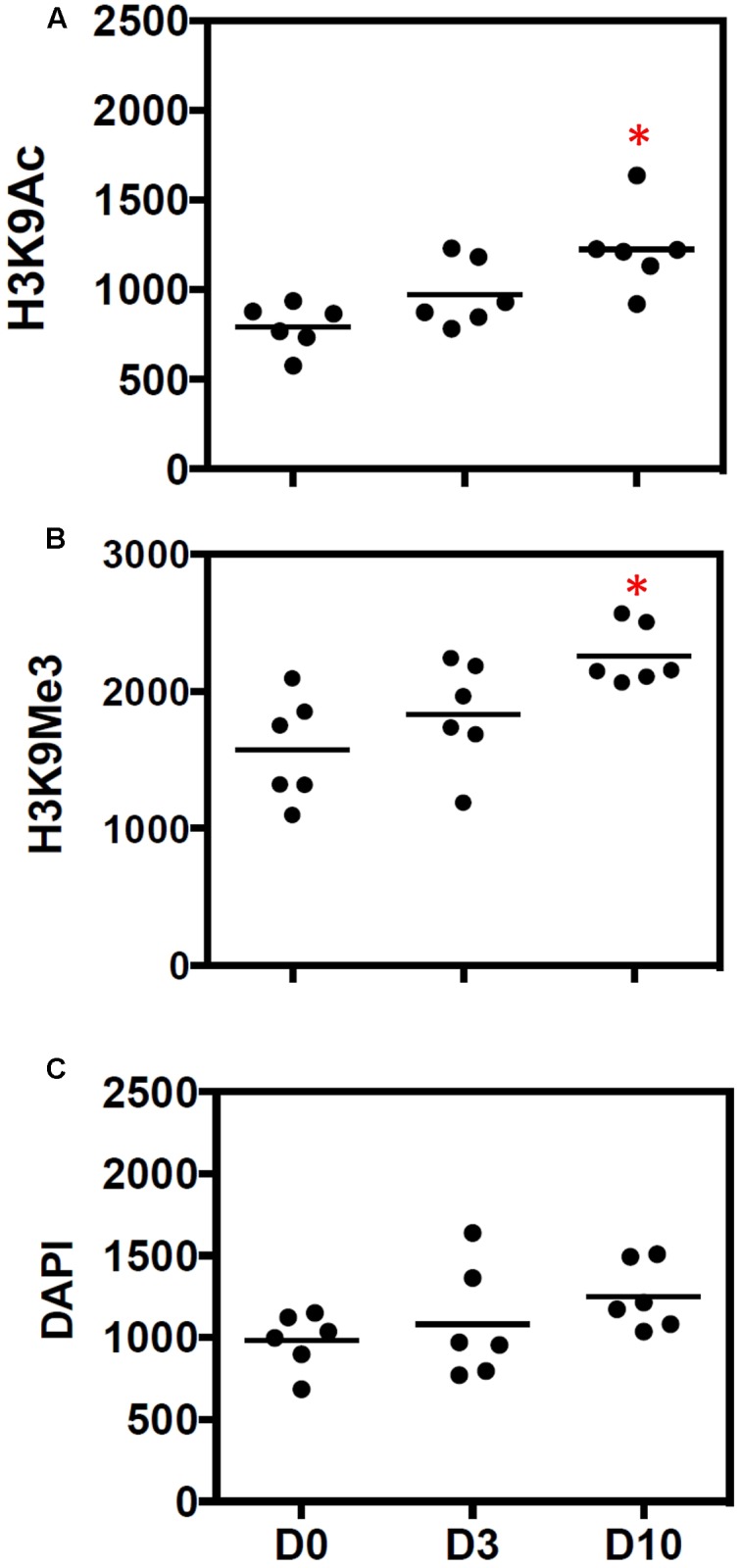
**Quantitative increase in histone marks during progressive fibrosis**. Figures show the number of **(A)** H3K9Ac and **(B)** H3K9Me3 marks before (D0) and after 3 days (D3) and 10 days (D10) of UUO. Results are expressed as number of individual histone marks per 0.25 mm^2^, with the average for each group shown with a bar. **(C)** DAPI staining was used to show the total DNA. *n* = 6 animals each group, ^∗^*p* < 0.05 vs. respective D0.

The average number of marks (per 0.25 mm^2^) was increased 10 days after UUO (**Figures 3A,B**). Enumeration of nuclei through DAPI staining showed no significant change in total cellularity/0.25 mm^2^ (**Figure 3C**).

### Expression of H3K9 Marks in Various Anatomical Compartments of the Kidney

**FIGURE 4:**
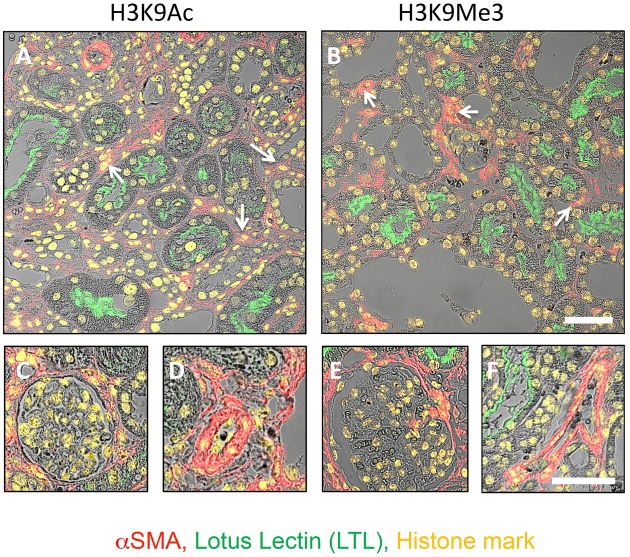
**Distribution of H3K9Ac and H3K9Me3 marks in the kidney after unilateral ureteric obstruction**. Combined confocal and brightfield microscopy of sections from an animal 10 days after UUO triple labeled for each histone mark (yellow), LTL (proximal tubule brush borders; green) and αSMA (myofibroblasts; red). Micrographs illustrate the distribution of each mark in the tubulointerstitium **(A,B)**, glomerulus **(C,E)**, and vasculature **(D,F**). Examples of histone mark positive myofibroblasts are shown with white arrows. Scale bar top panels = 50 μm, bottom panels = 25 μm.

Laser confocal microscopy at higher magnification and resolution was used to qualitatively characterize renal expression of the histone marks H3K9Ac and H3K9Me3 in the tubulointerstitium (**Figures 4A,B**), glomerulus (**Figures 4C,E**), and vasculature (**Figures 4D,F**). Combined confocal and brightfield microscopy of D10 sections with multiple labels showed the presence of H3K9Me3 marks in both proximal tubules (LTL lectin positive) and other tubules, and myofibroblasts (αSMA positive cells) (arrows **Figures 4A,B**). Both marks were expressed in glomeruli (**Figures 4C,E**). A merging of staining for each mark and αSMA showed that H3K9Ac and H3K9Me3 were also found in the vasculature (**Figures 4D,F**).

### TGF-β1 Activation of Rat Renal Fibroblast and NRK-52e *In Vitro*

TGF-β1 expression induced by UUO is known to play an important role in the development of renal fibrosis (Chevalier, 1996). Given the fundamental importance of TGF-β1 to fibrogenesis, we set out to use an *in vitro* model to further investigate the H3K9 specific effects of TGF-β1 in individual cell populations.

To do this we firstly confirmed that our fibroblast and tubule cell cultures were responsive to TGF-β1 (Supplementary Figures S1, S2). In fibroblasts, flow cytometric analysis of forward scatter (FSC) was used to measure changes in cell size. The right shift in FSC indicated that cells grown for 48 h in media supplemented with 1 ng/ml TGF-β1 (red) are larger than their untreated counterparts (black) (Supplementary Figure S1A). Consistent with this, confocal microscopy localized αSMA to stress fibers (arrows) immediately beneath the cell membrane, a defining characteristic of activated fibroblasts, so-called myofibroblasts (Darby et al., 1990) (Supplementary Figure S1B). Treatment with 1 ng/ml TGF-β1 for 48 h increased both the number of cells staining for αSMA (myofibroblasts), and the presence of stress fibers in individual cells (Supplementary Figure S1B). Similar characterization studies of the widely used immortalized renal tubule cell, NRK-52e, confirmed their proximal tubule origin (PHA-L positive) (Supplementary Figure S2A). Cells were also positive for vimentin (Supplementary Figure S2A), a cytoskeletal protein marker of dedifferentiation in epithelia (Loghman-Adham et al., 2003). Immunofluorescent microscopy (Supplementary Figure S2B) and Western blotting (Supplementary Figure S2C) showed that αSMA expression in NRK-52e were likewise responsive to 10 ng/ml TGF-β1 over 48 h, although this effect was much more modest than in fibroblasts. This was paralleled by a reduction in the epithelial cell junction protein E-cadherin (Supplementary Figures S2B,C).

### Histone Marks in Cell Cultures and Response to TGF-β1

**FIGURE 5:**
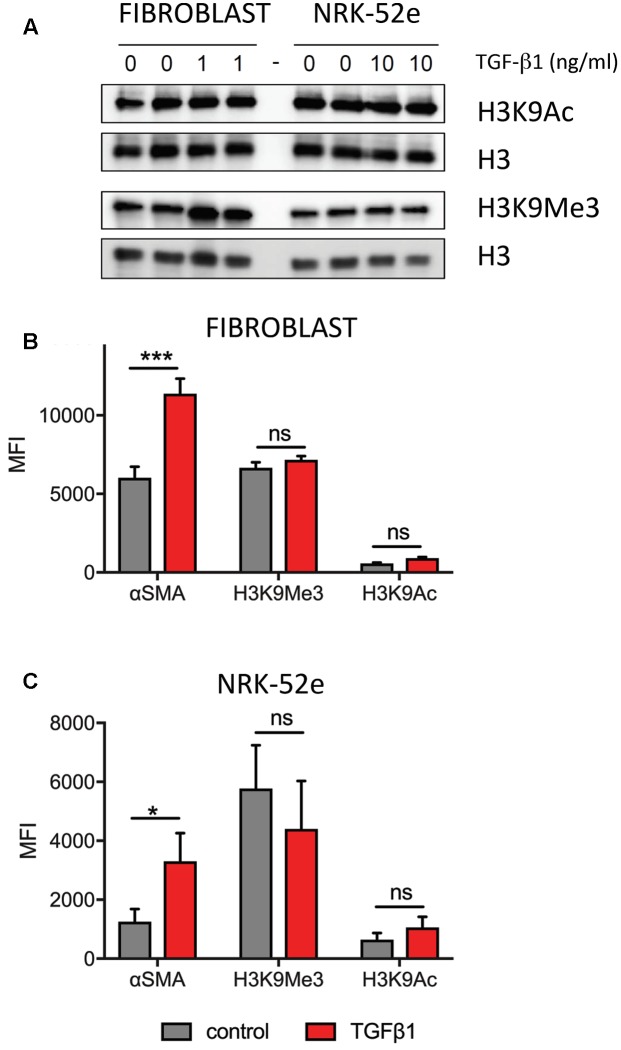
**Pooled analyses show no overall effect of TGF-β1 on H3K9 mark enrichment in (myo)fibroblasts and NRK-52e. (A)** Western blotting of duplicate samples from fibroblasts and NRK-52e with and without 48 h of TGF-β1 stimulation. Parallel blots were probed for H3K9Ac and H3K9Me3. Each blot was stripped and re-probed for H3 to correct for loading. **(B)** Summary of flow cytometric analysis presented as mean fluorescent intensity (MFI) of triplicate experiments in **(B)** fibroblasts and **(C)** NRK-52e. Bars represent mean ± SD from three independent experiments. ^∗^*p* < 0.05, ^∗∗∗^*p* < 0.001 vs. control; ns, not significant.

Western blotting established expression of both marks, which was unchanged by TGF-β1 treatment (**Figure 5A**). A similar analysis was undertaken using flow cytometry to quantitate each mark, with results expressed as a mean fluorescent intensity (MFI) of three independent experiments. In agreement with the Western blot analysis, the MFI of each mark did not change in either fibroblast (**Figure 5B**) or NRK52e (**Figure 5C**) after TGF-β1 stimulation, despite an almost doubling of mean αSMA intensity in each case.

### Quantitative Analysis of H3K9 Marks and Cell Phenotype

**FIGURE 6:**
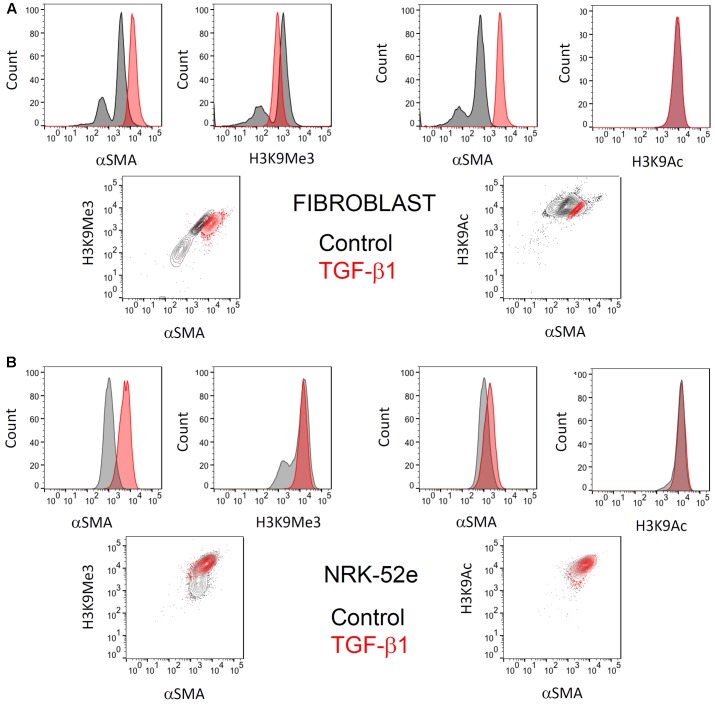
**Representative flow cytometric analysis comparing the effect of TGF-β1 stimulation on H3K9 marks and αSMA expression in (myo)fibroblasts and NRK-52e**. Flow cytometric analysis of H3K9Ac and H3k9Me3 modifications, and their relationship with **(A)** myofibroblast differentiation after 48hrs of treatment with 1 ng/ml TGF-β1 and **(B)** αSMA expression in NRK52e after 48 h of stimulation with 10 ng/ml TGF-β1. Normalized frequency histograms with the control cell group shown in gray and TGF-β1 stimulated cells plotted in red. Contour plots depict changes in double-staining.

When pooling all results, there was no overall change in intensity, but analysis of individual replicate experiments showed that the MFI masked underlying subtle changes in histone marks with treatment. As in our characterization studies, flow cytometry indicated that the fibroblast cultures used consisted of two distinct populations of cells with respect to αSMA expression (**Figure 6A**). Again in accordance with our characterization studies, TGF-β1 treatment produced a single homogenous population with increased αSMA expression.

Similarly, two distinct populations of H3K9Me3 positive cells existed. Treatment with exogenous TGF-β1 also produced a homogenous population of cells with H3K9Me3 expression, albeit intermediate in intensity to that seen in untreated (myo)fibroblasts (**Figure 6A**). On the other hand, the presence of H3K9Ac was more homogenous and did not change quantitatively with TGF-β1 treatment.

The αSMA response to TGF-β1 in NRK-52e was more modest, but significant (**Figure 6B**). Again however flow cytometry showed a heterogeneous population of H3K9Me3 marks, with TGF-β1 producing a population of cells with intermediate characteristic. TGF-β1 did not result in any enrichment of H3K9Ac marks.

### Sub-nuclear Distribution of Histone Marks after TGF-β1 Stimulation

As TGF-β1 seemed to induce more than simple enrichment of H3K9Me3, we used confocal microscopy to examine the sub-nuclear distribution of the two histone marks.

**FIGURE 7:**
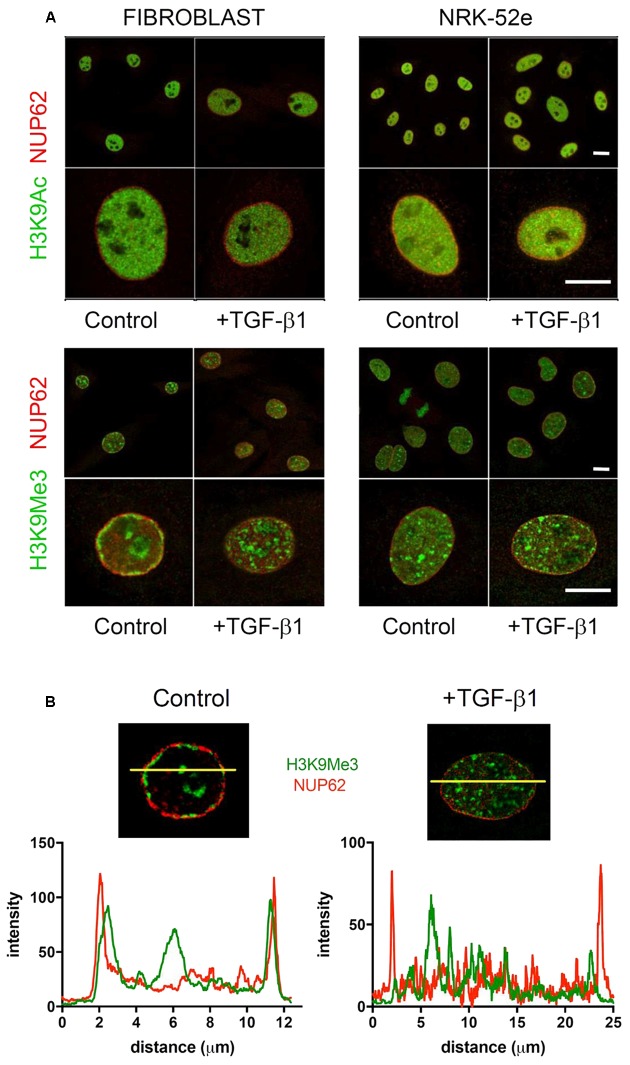
**Effect of TGF-β1 on the sub-nuclear distribution of histone marks. (A)** Confocal images of immunofluorescently stained nuclei from fibroblasts and NRK-52e incubated with and without exogenous TGF-β1. Merged confocal micrographs show relative positioning of histone marks (H3K9Me3, H3K9Ac) and the nuclear envelope protein nucleoporin 62 (NUP62) in cells grown in control media, and media supplemented with TGF-β1. Scale bars = 10 μm. **(B)** Co-localisation of staining intensity for H3K9Me3 and NUP62 at a central nuclear plane (yellow line) in a nucleus from each experimental group.

Confocal images of immunofluorescently stained (myo)fibroblast nuclei from cells incubated with and without exogenous TGF-β1 are shown in **Figure 7**. In control media, merged confocal micrographs show juxtapositioning of H3K9Me3 marks with the nuclear envelope protein nucleoporin 62 (NUP62) (**Figure 7A**). This relationship is graphically demonstrated in **Figure 7B** where the fluorescent intensity of each peaks at the nuclear periphery, with H3K9Me3 labeling adjacent but not overlapping with NUP62. Treatment with 1 ng/ml TGF-β1 resulted in a change in nuclear distribution of H3K9Me3, with loss of peripheral staining (**Figure 7B**). TGF-β1 stimulation had no effect on the distribution of H3K9Ac (**Figure 7A**).

**FIGURE 8:**
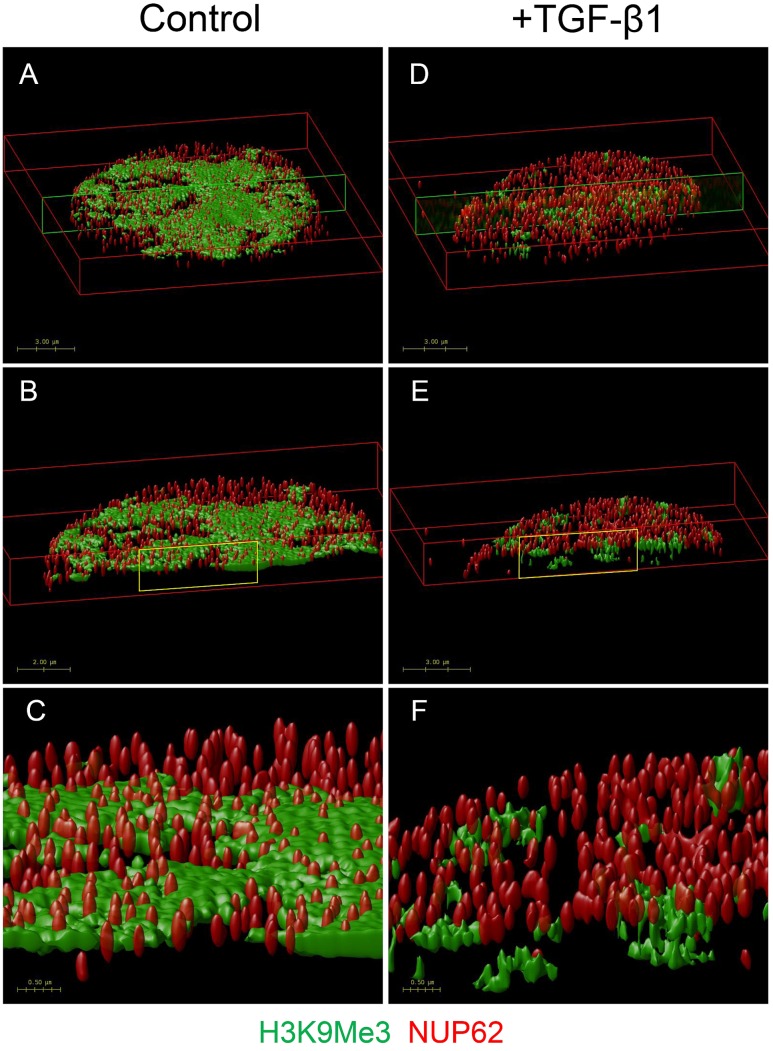
**OMX super-resolution microscopy demonstrating the effect of TGF-β1 on the nuclear distribution of H3K9Me3 marks**. Volume rendered images showing effect of TGF-β1 on distribution of H3K9Me3, and its relationship with the nuclear envelope. H3K9Me3 marks are shown in green with individual nuclear pores seen with red NUP62 staining. Refer to Supplementary Figure S3 for representative optical cross sections of immunofluorescent staining before image stacking and volume rendering. Figure shows the top 2 μm of nuclei from cells grown in **(A)** control media and **(D)** media supplemented with 1 ng/ml TGF-β1. A transverse cross section of each is indicated by the green box, and illustrated in **(B,E)**. A higher magnification of the areas marked in yellow **(C,F)** clearly shows the relative position of NUP62 and H3K9Me3 with and without TGF-β1 stimulation.

In an additional experiment, we used OMX blaze super-resolution microscopy to resolve the relationship of H3K9Me3 with the fibroblast nuclear envelope. Individual nuclear pores can be seen with NUP62 staining. TGF-β1 resulted in a loss of peripheral H3K9Me3 enriched chromatin (**Figure 8** and Supplementary Figure S3).

The intense staining for H3K9Ac in NRK-52e was diffuse and distributed evenly throughout most of the nucleus (**Figure 7A**). The distribution was qualitatively unchanged by TGF-β1 treatment. Unlike in fibroblasts, the H3K9Me3 marks were not preferentially associated with the nuclear membrane when grown in control media, and did not change with TGF-β1 stimulation (**Figure 7A**).

### Effect of TGF-β1 Stimulation on Cell Cycle Progression

**FIGURE 9:**
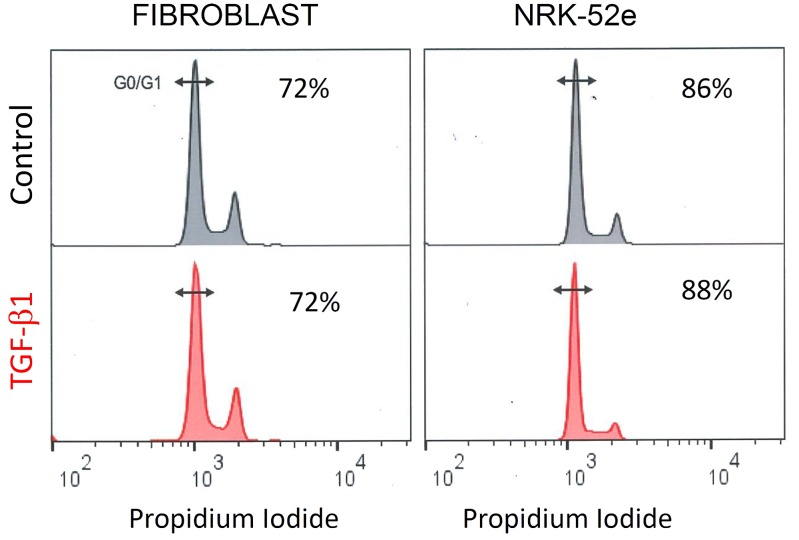
**TGF- β1 stimulation does not affect cell cycle progression in fibroblasts and NRK-52e**. Representative cell cycle analysis of propidium iodide stained fibroblasts and NRK-52e epithelial cells. The cell populations were analyzed for DNA content after propidium iodide staining using flow cytometry. Percentage of cells in the G0/G1 phase for each group are indicated.

Propidium iodide staining showed that TGF-β1 did not affect the proportion of cells in G0/G1 in either cell type. When grown in control media, flow cytometry showed that 72% of fibroblasts, and 86% of NRK-52e, were in G0/G1 phase. Parallel groups treated with TGF-β1 had 72 and 88% of cells in G0/G1 respectively (**Figure 9**).

### Sub-nuclear Localisation of Transcriptionally Permissive H3K9Ac and Transcriptionally Repressive H3K9Me3 during Fibroblast Activation

**FIGURE 10:**
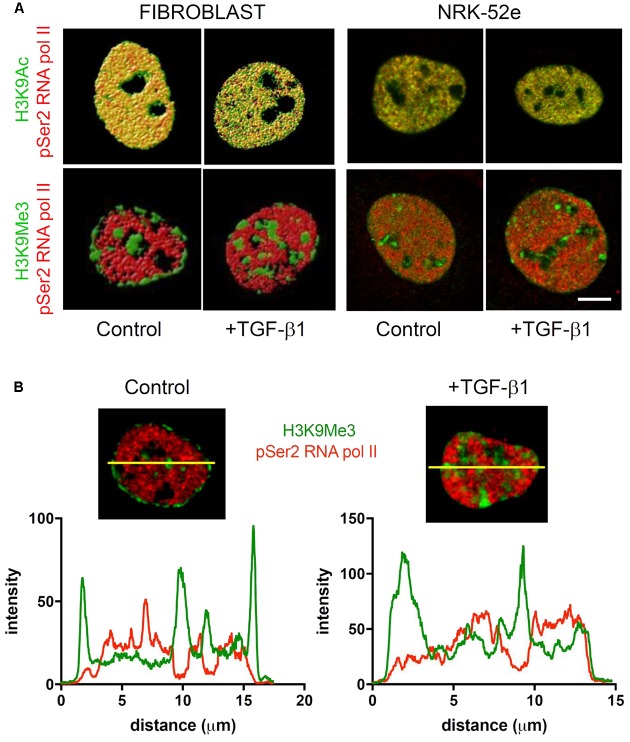
**Sub-nuclear localisation of transcriptionally permissive H3K9Ac and transcriptionally repressive H3K9Me3 in response to TGF-β1. (A)** Volume rendered confocal images of nuclei from cells incubated with and without exogenous TGF-β1. Merged micrographs compare localisation of H3K9Ac (green)/H3K9Me3 (green) with phosphorylated RNA polymerase II (pSer2 RNA pol II) (red) in fibroblasts and proximal tubules (NRK-52e). Yellow indicates co-localisation of mark and pSer2 RNA pol II. Scale bar = 5 μm. **(B)** Co-localisation of staining intensity for H3K9Me3 and pSer2 RNA pol II at a central nuclear plane (yellow line) in a nucleus from each group.

Rendered confocal images of fibroblast nuclei from cells incubated with and without exogenous TGF-β1 are shown in **Figure 10**. Merged micrographs show co-localisation (yellow) of H3K9Ac (green) and phosphorylated RNA polymerase II (pSer2 RNA pol II) (red), a marker of active transcription. Conversely, localisation of H3K9Me3 and pSer2 RNA pol II are quite distinct, which was confirmed in an analysis of staining intensity (**Figure 10B**). As above, treatment with TGF-β1 had no effect on the distribution of H3K9Ac but resulted in changes in the spatial distribution of H3K9Me3. There was a parallel loss of peri-nuclear membrane marks, and an increase in H3K9Me3 enriched chromatin within the nucleus, in compartments that resembled the nucleolus.

Again the diffuse staining for H3K9Ac (green) and pSer2 RNA pol II (red) were co-localized (yellow) in NRK-52e nuclei, which was unchanged by TGF-β1 (**Figure 10A**). As seen previously, H3K9Ac was not preferentially localized to the nuclear periphery or compartmentalized in these cells, and was unchanged with TGF-β1 stimulation (**Figure 10A**).

## Discussion

In this study, we have examined the distribution and acquisition of H3K9 modifications in response to injury and TGF-β1 stimulation. Principal findings are that the histone marks H3K9Ac and H3K9Me3 are widely expressed in the normal kidney, with a small quantitative change during progressive fibrosis. *In vitro*, TGF-β1 caused a remodeling of the nuclear landscape, that was both cell and mark dependent but had no overall effect on mark enrichment.

A growing body of evidence suggests that epigenetic regulation is important in renal fibrogenesis. Most attention to date has focused on the role of non-coding micro RNA (miRNA) (Gomez et al., 2016) and DNA methylation (Bechtel et al., 2010). Less well-understood however is the role of histone modifications in this process.

How the cell deciphers the multitude of modifications that constitute the histone code has long been controversial, although most available evidence now suggests that both direct and indirect models are involved (Taverna et al., 2007; Greer and Shi, 2012). Acetylation can directly affect chromatin compaction by neutralizing the interaction between basic histones and negatively charged DNA to relax or de-condense chromatin (euchromatin), thereby increasing access for transcription machinery. In the indirect model, histone marks are read by protein effector modules termed readers, which alter transcription by recruiting various other molecules to either stabilize chromatin or facilitate transcription (Bannister et al., 2001).

To our knowledge, most contemporary studies have examined aggregate changes in histone marks using Western blotting techniques, with few studies examining the *in vivo* distribution of marks. Those that do, have not reported findings in the normal kidney (Marumo et al., 2010). The presence of both H3K9 marks in the normal kidney was more ubiquitous than we expected, although they were not found in every cell. The higher resolution offered by confocal microscopy was used to characterize tubulointerstitial distribution of marks after injury. Both H3K9Ac and H3K9Me3 were diffusely expressed in interstitial myofibroblasts, and tubule epithelial cells, including, but not limited to, proximal tubules. The quantitative changes after UUO were relatively modest, but significant and independent of changes in overall cellularity. Nonetheless, these quantitative *in vivo* changes do however suggest that progression of fibrosis involves divergent changes in gene expression regulation given their reciprocal action on transcription.

*In vitro*, untreated cultures consisted of a single population of H3K9Ac expressing cells by flow cytometry, the diffuse staining pattern of H3K9Ac in both fibroblasts and epithelial cells being consistent with the diffuse distribution of transcriptionally active euchromatin (Reddy and Natarajan, 2011). Conversely, both fibroblast and epithelial cultures contained heterogeneous populations of H3K9Me3 expressing cells. However, the nuclear distribution in each was different with H3K9Me3 localized to the nuclear sub-compartments in both fibroblasts and tubule epithelial cells, and in the case of fibroblasts, also adjacent to the nuclear membrane. Transcriptionally silent heterochromatin tends to be positioned at the nuclear periphery (Andrulis et al., 1998), with stepwise methylation of histone H3K9 emerging as one pathway of assembly and heterochromatin-induced gene silencing (Towbin et al., 2013). Accordingly, we were able to co-localize H3K9Me3 with the nuclear pore protein NUP62.

In agreement with the transcriptionally permissive and repressive role of each mark (Barski et al., 2007; Koch et al., 2007), H3K9Ac was co-localized with pSer2RNA polymerase II (pRNApol II), a marker of transcriptional elongation and therefore active mRNA transcription machinery (Ghamari et al., 2013), whereas, H3K9Me3 loci were distinct from staining for pRNApol II and therefore not associated with active mRNA transcription. Fibrogenesis is associated with divergent and complex spatiotemporal changes in gene expression, and not simply a generalized upregulation of pro-fibrogenic transcripts. The rearrangement of marks seen here is consistent with our recent gene expression profiling study of TGF-β1 signaling in these cells, where TGF-β1 stimulated the concomitant activation of some genes and suppression of others (Smith et al., 2017). Further work is required to determine which genes are altered by the subtle quantitative and spatial epigenetic modifications identified here as well as the cell-specific significance of such changes.

Flow cytometry suggested that there were underlying changes in epigenetic states not discernible at the pooled level. In fibroblasts, a more detailed analysis of this data showed that H3K9Ac marks were uniformly expressed, were unchanged by TGF-β1 stimulation, and were unrelated to myofibroblast differentiation. Conversely, TGF-β1 seemed to result in a synchronization of cells to a homogenous population with respect to H3K9Me3 staining, which clearly correlated with myofibroblast differentiation (αSMA acquisition). Interestingly this did not involve simple enrichment, or loss of marks, as staining intensity was intermediate. Super-resolution microscopy revealed further qualitative effects of TGF-β1 on H3K9Me3, showing dissociation of this mark from the nuclear membrane. Importantly, these changes in distribution were not secondary to changes in mitotic activity or cell cycle progression, as both were unaltered by TGF-β1 treatment. Taken together, these findings in the fibroblast capture hitherto unrecognized changes in H3K9Me3 nuclear dynamics induced by TGF-β1, that are not apparent using aggregative quantitative analyses.

For many years the prevailing dogma was that histone methylation marks were inherently more stable and permanent than their acetylation counterparts (Bannister et al., 2001). Our observations may provide an additional insight if the redistribution seen represents a simultaneous demethylation of existing marks and *de novo* acquisition in other nuclear compartments, rather than simply a relocation of marks to a different compartment.

The apparent increased enrichment in more interior sub-nuclear compartments is also of particular interest. The size, distribution, lack of membrane, and lack of mRNA transcription in these compartments suggest that they are likely to be nucleoli. It will be interesting to see if there is a specific role for H3K9Me3 in regulating ribosome function in these cells during fibrogenesis (Drygin et al., 2010), similar to how nucleolar enrichment of methylation marks is regulatory in liver cancer (Yu et al., 2015).

Our studies also examined the effect of TGF-β1 stimulation of NRK-52e. Notwithstanding that these cells may have already been in a transitional state, as indicated by the constitutive expression of αSMA and vimentin (Franke et al., 1979; Loghman-Adham et al., 2003; Doi et al., 2011), the distribution of H3K9Ac was homogeneous, and quantitatively unchanged after TGF-β1 stimulation. Two populations of H3K9Me3 cells existed in control cultures, with TGF-β1 exposure again resulting in a single homogenous culture with intermediate H3K9Me3 expression as in the fibroblast. The molecular nature of this synchronization in both cell types awaits elucidation.

Although beyond the scope of this work, future studies will need to examine the downstream molecular and functional effects of these structural modifications, the enzymes involved, and their relationship with TGF-β1 signal transduction. Nevertheless, our findings provide evidence of the dynamic state of H3K9 modification during renal fibrogenesis, and in particular, complex spatial changes in the nuclear distribution of H3K9Me3 induced by TGF-β1 that go beyond simple quantitative augmentation. Given the fundamental importance of the myofibroblast and TGF-β1 to fibrogenesis, these findings are likely to have implications for our understanding of the response to injury in the kidney.

## Author Contributions

Participated in research design, acquisition, analysis and interpretation: TH, SH, S-JT, BW, CS, ES. Wrote or contributed to writing of manuscript: TH, SH, S-JT, BW, CS, ES. Have given final approval: TH, SH, S-JT, BW, CS, ES. Agree to be accountable for the work: TH, SH, S-JT, BW, CS, ES.

## Conflict of Interest Statement

The authors declare that the research was conducted in the absence of any commercial or financial relationships that could be construed as a potential conflict of interest.
