# Supplementary material for: Epigenetic Modifications to H3K9 in Renal Tubulointerstitial Cells after Unilateral Ureteric Obstruction and TGF-β1 Stimulation
Source: Front Pharmacol. 2017 May 29;8:307. doi: 10.3389/fphar.2017.00307 (PMC5447091; doi:10.3389/fphar.2017.00307)

frontiers

Supplementary Material

Epigenetic Modifications to H3K9 in Renal Tubulointerstitial Cells after Unilateral Ureteric Obstruction and TGF-β1 Stimulation

Timothy D Hewitson[1,2*], Stephen G Holt[1,2], Sven-Jean Tan[1], Belinda Wigg[1], Chrishan S Samuel[3], Edward R Smith[1,2]

*** Correspondence:** Timothy D Hewitson: tim.hewitson@mh.org.au

Figure s1. Cell characteristics and effect of TGF-β1 on rat renal fibroblast phenotype *in vitro*. (A) Comparative flow cytometric analysis of cell size using forward scatter (FSC) as a function of side scatter (SSC) in a population of (myo)fibroblasts grown in control media (black) and media supplemented with 1ng/ml of TGF-β1 (red). (B) Sub-cellular examination of αSMA expression in control cultures and cells treated with TGF-β1. White arrows show αSMA localized in stress fibres immediately beneath the cell membrane. Scale bar = 25μm.

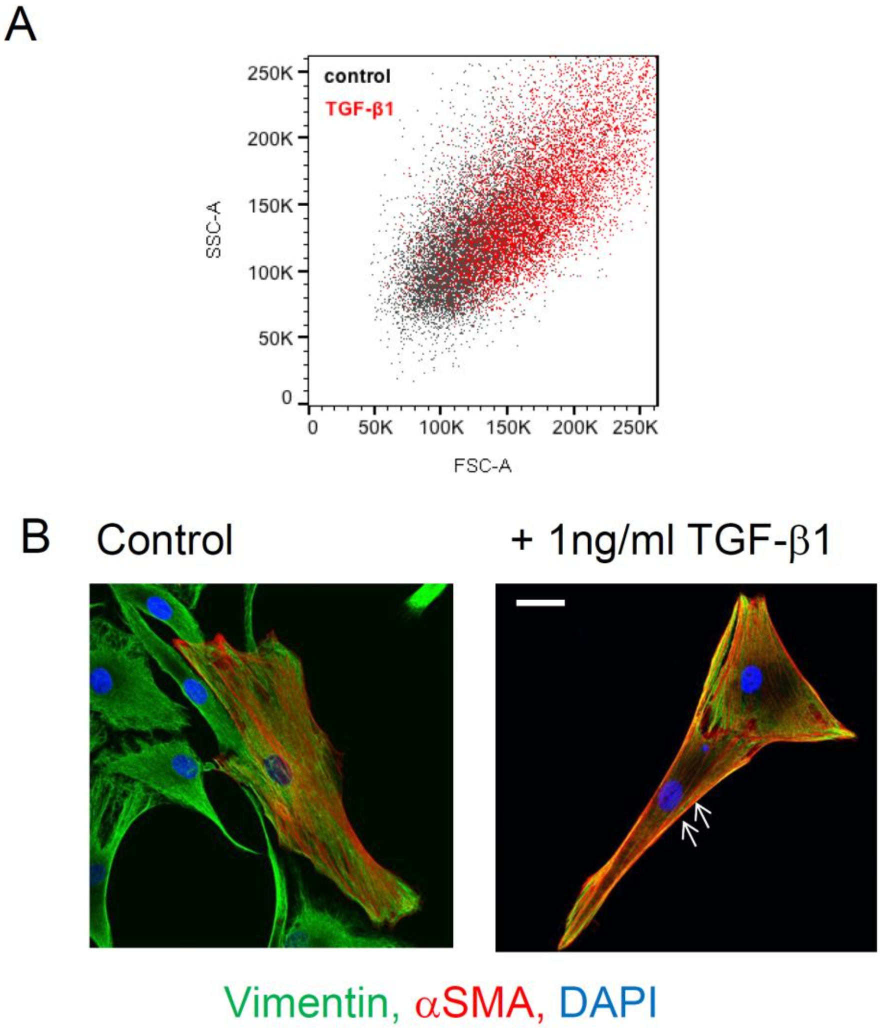

Figure s2. Cell characteristics and effect of TGF-β1 on NRK-52e phenotype *in vitro*. (A) Immunoperoxidase staining of confluent NRK-52e for the proximal tubule specific lectin Phaseolus haemaggutinin-l (Pha-L) and the cytoskeletal protein vimentin. Cell nuclei are counterstained with haematoxylin. (B) Immunofluorescent staining showing the effect of TGF-β1 stimulation on αSMA (Alex Fluor 594; red) and E-cadherin (Alex Fluor 488; green) expression in cultured cells. In each case cell nuclei are shown with DAPI (blue) staining. (C) Western blotting of triplicate samples showing effect of 10ng/ml TGF-β1 on E-cadherin and αSMA protein expression. A parallel blot was probed with β-tubulin to confirm equivalent loading. Scale bar = 100 μm.

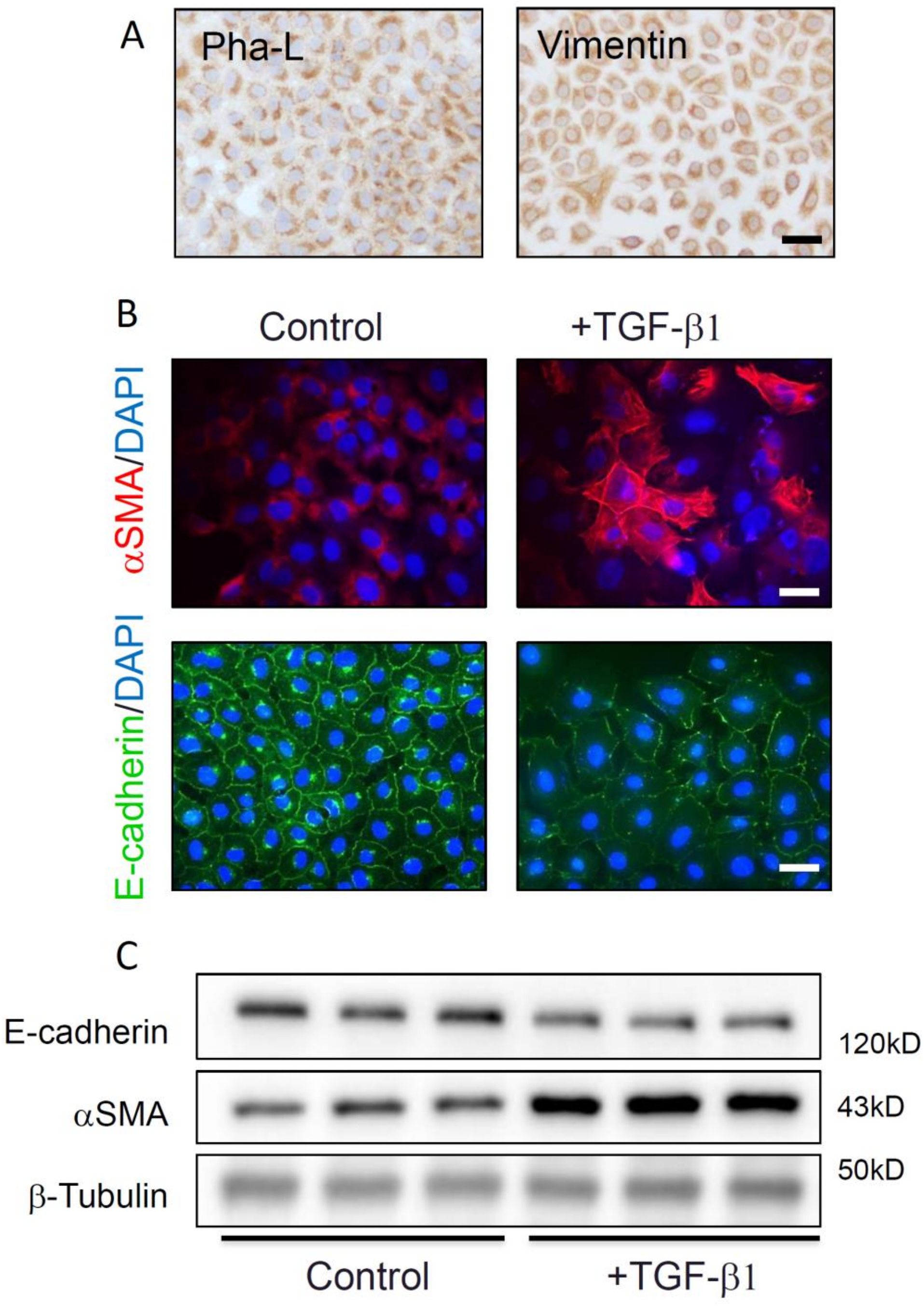

Figure s3. OMX super-resolution microscopy demonstrating the effect of TGF-β1 on the distribution of H3K9Me3 marks. Single representative optical sections from cells treated with control media, or TGF-β supplemented media, and simultaneously labelled for H3K9Me3 and the nuclear pore protein NUP62. The higher resolution of OMX allows the identification of individual pores. Scale bar = 5μm.

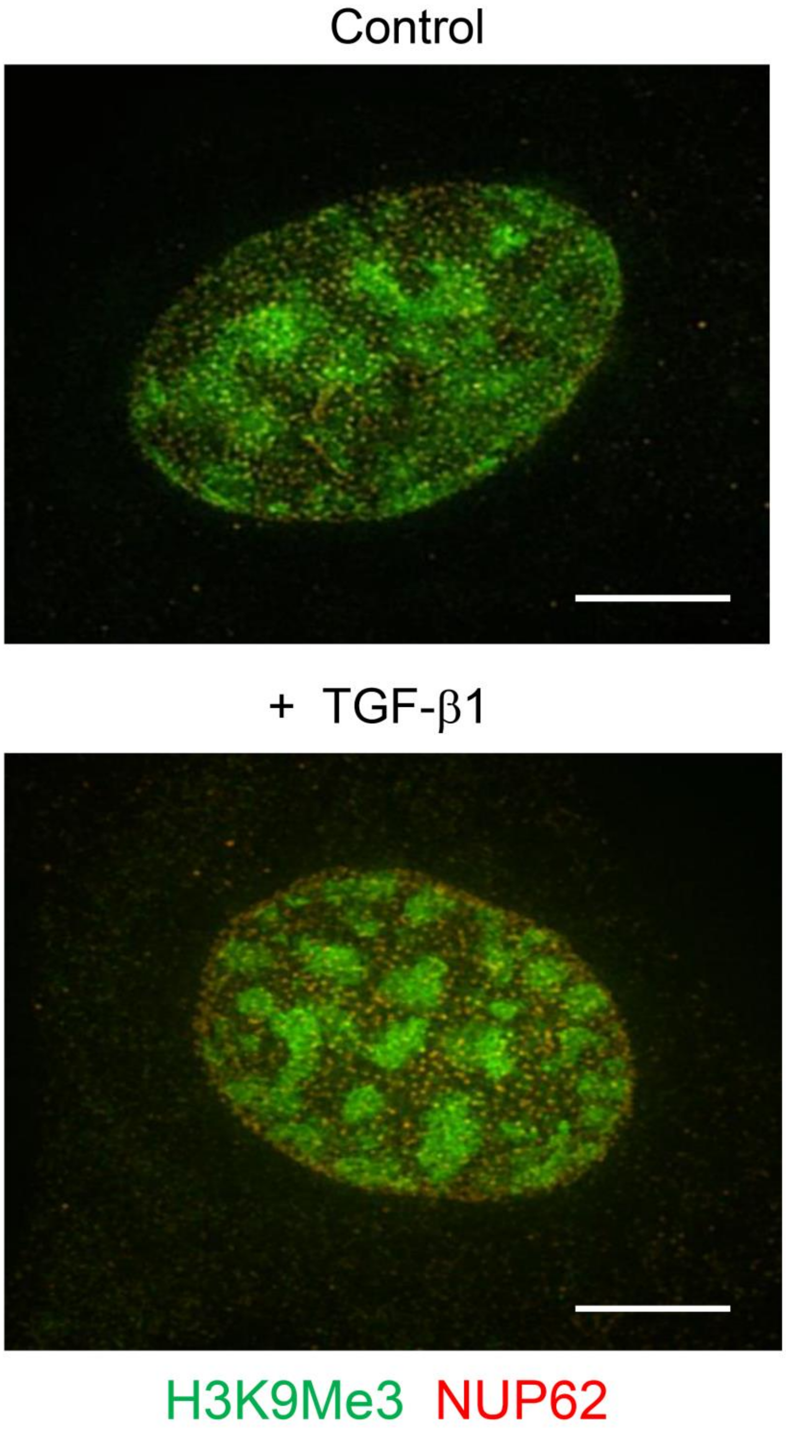

Supplement: Supplementary file 1 [file Presentation_1.PDF]
